# Astrocyte-Neuron Signaling in Synaptogenesis

**DOI:** 10.3389/fcell.2021.680301

**Published:** 2021-07-02

**Authors:** Lili Shan, Tongran Zhang, Kevin Fan, Weibo Cai, Huisheng Liu

**Affiliations:** ^1^Guangzhou Laboratory, Guangzhou, China; ^2^Bioland Laboratory (Guangzhou Regenerative Medicine and Health Guangdong Laboratory), Guangzhou, China; ^3^Department of Radiology, University of Wisconsin–Madison, Madison, WI, United States; ^4^Department of Medical Physics, University of Wisconsin–Madison, Madison, WI, United States

**Keywords:** astrocyte, synaptogenesis, neuron, human induced pluripotent stem cells, molecular signaling

## Abstract

Astrocytes are the key component of the central nervous system (CNS), serving as pivotal regulators of neuronal synapse formation and maturation through their ability to dynamically and bidirectionally communicate with synapses throughout life. In the past 20 years, numerous astrocyte-derived molecules promoting synaptogenesis have been discovered. However, our understanding of the cell biological basis underlying intra-neuron processes and astrocyte-mediated synaptogenesis is still in its infancy. Here, we provide a comprehensive overview of the various ways astrocytes talk to neurons, and highlight astrocytes’ heterogeneity that allow them to displays regional-specific capabilities in boosting synaptogenesis. Finally, we conclude with promises and future directions on how organoids generated from human induced pluripotent stem cells (hiPSCs) effectively address the signaling pathways astrocytes employ in synaptic development.

## Introduction

Glia got their name in 1856 when Rudolf Virchow observed a connective structure that seems to hold neurons together. He simply termed it “nervenkitt” (derived from Greek), which means nerve glia or nerve glue. Despite this discovery, glia received little attention among researchers until Santiago Ramón y Cajal’s histological study in the late 19th century, when he developed specific staining methods for glial cells and described their morphology. Based on his studies, in 1909, he raised the question: “What is the function of glia?” in 1909 (Garcia-Marin et al., 2007). Since then, significant attention has been brought upon discovering glia’s diverse physiological roles.

Glia encompass the largest population of the brain apart from their neuron neighbors, with the ratio of glia to neurons positively correlating to an animal’s brain size (Friede, 1963). As evolution has proceeded, glia and neurons have undergone substantive increases in cell number, diversity, and function, increasing brain complexity. This co-development of glia and neurons hints that the increasing number of glia could result from greater demands involved in synapse regulation. Throughout the entire central nervous system (CNS), glia are interspersed with neurons and perform a wide variety of pivotal functions during development and adulthood. Classically, it has been admitted that glia passively provide structural, trophic, and metabolic supports to neurons. However, recent studies have proved otherwise, revealing how glia play more active roles than previously thought. For example, glia-secreted factors promote synapse maturation and maintenance, orchestrate synaptic activity and neural circuit function (Allen, 2014). Furthermore, their fine processes increase synaptic receptivity (Barker et al., 2008) and stability (Bernardinelli et al., 2014).

This review discusses the role glia play in regulating synaptogenesis within the CNS, focusing on astrocytes, the most abundant subtype of glia in the CNS that compose ∼20–40% of total cells of the mammalian brain (Herculano-Houzel, 2014). Moreover, this review highlights promising studies on the application of astrocytes in human induced pluripotent stem cells (hiPSCs) to accelerate neurodevelopmental processes of hiPSCs-derived neurons, focusing on the astrocytes’ ability in neuronal differentiation and functional maturation.

## Astrocyte as a Component of the Whole Synapse

Synapses are the fundamental units in building the entire neural network, through which signals are transferred from pre-synaptic neurons to post-synaptic cells in a fast and point-to-point way (Sudhof, 2018). Specifically, in a chemical synapse, the pre-synaptic terminal releases neurotransmitters which then bind to highly selective receptors located on post-synaptic dendrites. In the early 1990s, Araque et al. (1999) found that glia intimately regulate and respond to neuronal activity and neurotransmission, which subsequently led to the term “tripartite synapses.” A series of studies have shown that neuron activity can induce intracellular long-duration calcium spikes and calcium waves in astrocytes, which subsequently trigger them to release chemical transmitters, leading to feedback inhibition of synaptic transmission and neuronal activity (Araque et al., 1999; Durkee and Araque, 2019). Neuronal glutamate can trigger the release of astrocyte-derived “gliotransmitters,” including D-serine, glutamate, or adenosine triphosphate (ATP). Astrocytes can respond to neurotransmitters, which are largely dependent on the membrane expression of astrocytic G protein-coupled receptors (GPCRs) (Kofuji and Araque, 2020), which can bind neurotransmitters and trigger intracellular calcium elevations (Agulhon et al., 2008). Observation showed that the kinetics of calcium signals in astrocytes are much slower compared to neurons (Durkee et al., 2019). Presumably, calcium rise in astrocytes is mainly mediated by GPCRs, while in neurons, it is primarily induced by ion channels. It is essential to mention that controversy exists in the neuroscience community, with terms including “tripartite synapses” and “gliotransmitter” being deemed “glio-centric.” A neutral point of view in terms of astrocytes’ function is that astrocytes provide an additional layer of information processing to synapses (Hamilton and Attwell, 2010).

**FIGURE 1:**
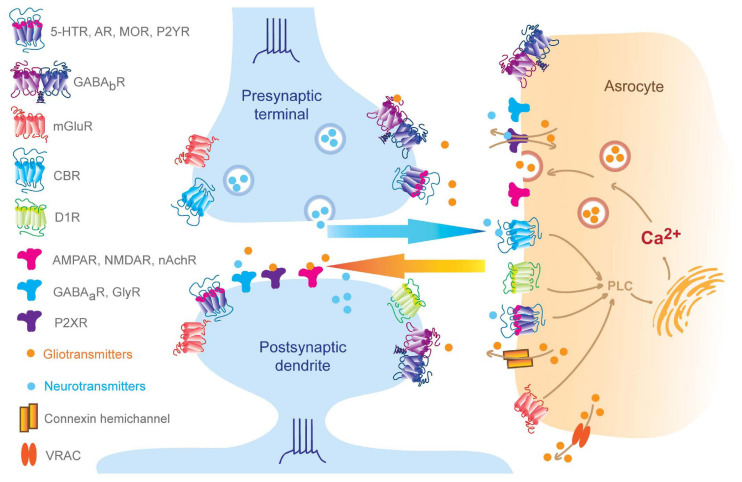
Bidirectional neuron-astrocyte signaling. Pre-synaptic neurotransmitters and post-synaptic release endogenous cannabinoids that bind to astrocytic GPCRs and ion channels, which result in elevation of intra-astrocyte calcium concentration and trigger gliotransmitter releasing, in turn, perisynaptic gliotransmitters bind to synaptic receptors regulating synaptic behaviors (Volterra and Meldolesi, 2005; Hamilton and Attwell, 2010; Benarroch, 2016; Kofuji and Araque, 2020). Of note, subset of AMPA receptors can be found at pre- and post-synaptic membrane and also astrocytes, such as GluR2 in the cerebellum (Matsuda et al., 2000; Rossi et al., 2008; Molders et al., 2018).

Besides the tight bidirectional chemical communications between astrocytes and neurons (see Figure 1), morphologically, a single astrocyte infiltrates its fine processes into neuropils and intimately wraps up to 140,000 synapses (Bushong et al., 2002). In turn, for each neuron, its synapses can be wrapped by numerous fine processes from different astrocytes. During the wrapping processes, neurons and glia undergo coordinated morphogenesis in minutes, with a size scale ranging from nanometers to hundreds of microns (Lamkin and Heiman, 2017). This coordination between neurons and glia is further supported by rodent brain slice studies that observed astrocyte processes and dendritic spines growing or shrinking together upon stimulation (Haber et al., 2006). Remarkably, these morphological changes are exquisitely restricted in space, which can be specific to a single spine. For example, photoactivation of an astrocyte process on a single synapse displays rapid actin-dependent movements, with motility changes only occurring in the astrocytic process at that synapse, with other nearby glia-spine interactions remaining unaffected (Bernardinelli et al., 2014). Pre-synaptic stimulation also leads to increased motility in the astrocytic process associated with its post-synaptic spine. Thus, both neuronal activity and astrocytic activation modulate perisynaptic astrocytic process motility. From these studies, questions regarding how glia-neuron attain this reciprocal association have been raised. Works in *Caenorhabditis elegans* might provide insights into the underlying mechanism coordinating the relationship between astrocytic processes and the dendritic spine. For example, amphid glia control amphid neurons’ shape through potassium chloride cotransporter 3 (KCC-3) and receptor-type guanylate cyclase gcy-8 (GCY-8) (Singhvi et al., 2016). KCC-3 localizes specifically to a glial microdomain surrounding amphid neuron microvilli, where it controls potassium and chloride concentration. The elevated chloride level, in turn, activates GCY-8 on neuron microvilli and produces cyclic guanosine monophosphate (cGMP) to inhibit microvilli shape through neuronal wiskott–Aldrich syndrome protein and cyclic nucleotide-gated channels. Notably, the astrocyte-neuron coordinating maturation was observed especially during the second and third post-natal weeks, a time window when astrocytes complete duplication and become capable of communicating with synapses (Freeman, 2010). Of note, this temporal relationship indicates other mechanisms may exist. Zehnder et al. (2021) discovered that there is a transient high expression of astrocytic mito oxidative metabolism relevant genes, such as mitochondrial biogenesis genes (*PGC-1*α and *PGC-1*β), and oxidative phosphorylation genes (*Cox5b*, *Cox41l*, *Atp5a1*, and *Cycs*) during the third week following birth. Accordingly, astrocytes contain more ATP content and consume more oxygen during the third post-natal week compared to day 50. Conditional astrocytic peroxisome proliferator-activated receptor gamma coactivator 1 (PGC-1) knockout (KO) dampens cortical astrocyte maturation, including increased proliferation, smaller domains, fewer branches, and less distance between somas. In line with this, the deletion of astrocytic metabotropic glutamate receptor (mGluR) 5, a gene exhibiting a transient high expression level during the first post-natal week (Cahoy et al., 2008; Zhang et al., 2016), leads to aberrant mitochondrial distribution and smaller size as well as impaired astrocyte and neuron maturation, suggesting a causal link between astrocytic mGluR5 and the coordination of astrocyte-neuron maturation. This also reinforces the idea of a possible mechanism that neurons communicate with astrocytes through mGluR5, triggering astrocytic mitochondrial biogenesis that results in the expression of increased ATP release and, consequently, promotion of the formation of neighboring synapses. Taken together, these morphological and functional properties of astrocytes provide the ability to sense, integrate and regulate information among their enwrapped synapses, thus working as a component of the whole synapse.

## Glia Promote Synaptogenesis

Synaptogenesis has traditionally been regarded as a process of synapse formation solely involving neurons (Haydon and Drapeau, 1995). During synapse formation, but before physical neuron-neuron interactions, neurons are intrinsically programed to synthesize pre- and post-synaptic components, allowing *trans*-synaptic cell-adhesion molecules (CAMs) to bidirectionally orchestrate synapse formation through the assembly of pre-existing synaptic components during physical contact with CAMs (Bukalo and Dityatev, 2012). However, descriptive studies designate a potential role of astrocytes in synaptogenesis. Synaptophysin is a synaptic vesicle protein with its immunoreactivity studied in human postmortem brain tissues as a marker for maturation in the fetal brain (Sarnat et al., 2010). In the hippocampus, synaptophysin became visible in the dentate gyrus at 12 weeks, followed by CA2 at 14 weeks, CA3 at 15 weeks, CA4 at 16 weeks, and cornu ammonis (CA) 1 at 19 weeks, suggesting a precise spatiotemporal maturation of synaptic components. Indeed, the sequence of synaptogenesis happens in a variety of structures in the developing human fetal brain (Sarnat et al., 2010, 2013a,b). Interestingly, the hippocampal neuron maturation time window just falls behind the astrocyte appearance starting after 14 weeks of gestation (Stagaard Janas et al., 1991a,b). This correlation calls into question the role astrocytes play in synaptogenesis. In the past 20 years, a steady stream of works has demonstrated that astrocytes actively promote synaptogenesis (Freeman and Rowitch, 2013; Khakh and Sofroniew, 2015; Wilton et al., 2019). Using a two-step “panning” procedure, Barres et al. (1988) can get nearly 100% purity of retinal ganglion cells (RGCs), discovering that these highly purified neuron cultures could survive but grew inefficient synapses. Flowing the addition of colliculus neuroglia to cultures, RGCs achieved significantly enhanced synapse maturity compared to pure RGC’s (Pfrieger and Barres, 1997; Ullian et al., 2001). Immunochemical staining and electron microscope imaging data demonstrated that glia conditioned medium (GCM) treatment dramatically increases the number of synapses to nearly seven times in RGCs cultures (Nagler et al., 2001; Ullian et al., 2001). Thus, glia or glia-conditioned medium can both directly accelerate synaptogenesis, indicating that glia-secreted factors might be the underlying cause for this process. Glia-derived factors that facilitate synaptogenesis will be further reviewed in the following sections.

## Identification of the First Glia-Derived Synaptogenic Factor–Cholesterol

Brain lipids are highly demanding components for the assembly of neuron structure and machinery. Cholesterol, in particular, is not only an essential structural component of the lipid bilayer but also a highly enriched component within synaptic vesicles and synaptosomes (Goritz et al., 2005; Pfenninger, 2009). In terms of cholesterol metabolism, the brain is considered an autonomous entity, given the difficulty of penetrating the blood-brain barrier. Thus, brain cholesterol supply is primarily dependent on *de novo* synthesis (Bjorkhem and Meaney, 2004). Seminal experiments have shown that neurons can synthesize a sufficient amount of cholesterol to survive, grow, and form inefficient synapses, but must uptake additional astrocytic cholesterol for the formation of massive functional synapses (Pfrieger and Barres, 1997; Mauch et al., 2001; Ullian et al., 2001). The first evidence suggesting that cholesterol is the active component in GCM was performed on RGC cultures. *In vitro* experiments found that adding cholesterol to glia-free RGC cultures increased the frequency of spontaneous excitatory post-synaptic currents to more than ten times, which was comparable to GCM. Lowering the cholesterol concentration in GCM blocks GCM-induced enhancement of synaptic activity, which can be fully restored by additional cholesterol (Mauch et al., 2001). In cholesterol absent single-neuron microcultures, most microtubule-associate protein 2 (MAP2), a neuron dendrite specific marker, accumulates in the soma, with only 20% of dendrites harboring MAP2. In contrast, the presence of cholesterol leads to a greater increase of neurotic cholesterol content and the number of MAP2-positive dendrites, indicating that dendritic development is the rate-limiting step for cholesterol-induced synaptogenesis. Accordingly, increased GluR2/3 expression and evoked excitatory post-synaptic currents were also observed in both GCM and cholesterol-containing conditions, while the absence of cholesterol eliminates GluR2/3 and dendrites (Craig et al., 1993; Goritz et al., 2005). Taken together, these pieces of evidence demonstrated that glia-derived cholesterol promotes synapse formation and functional maturation.

How does glia deliver cholesterol to neurons? Apolipoprotein E (apoE) has been identified when comparing RGC membrane composition between culture conditions with and without glia by mass spectrometry (Mauch et al., 2001). Apolipoproteins are components of different lipoproteins that form lipoprotein particles and transport lipids. Astrocytic apoE and cholesterol can be complexed to apoE containing lipoproteins, and then be recognized by neuron apoE receptors, such as the light-density lipoprotein (LDL) receptor (Mahley, 2016). Interestingly, additional glia-derived apoE gives no synaptogenesis effect, although apoE plus cholesterol lead to significant synaptogenesis. This finding suggests that apoE is only responsible for neuron-glia cholesterol delivery, and neuron-glia lipid metabolism is vital for supporting synaptogenesis properly.

## The Role of Neuro-Glia Metabolism in Synaptogenesis

*In vivo* experiments have demonstrated that astrocyte lipid metabolism modulates pre-synaptic terminal maturation and post-synaptic receptor clustering and stability as well as dendritic arborization. The sterol regulatory element-binding protein 1 (SREBP-1) is a basic helix-loop-helix leucine zipper (bHLH-Zip) transcription factor, which regulates the synthesis and cellular uptake of cholesterol and fatty acids. In the hippocampus, SREBP-1 is expressed by astrocytes and can be activated by SREBP cleavage-activating protein (SCAP), especially during lipoprotein assembly (Madison, 2016). SCAP depletion in the hippocampal astrocytes severely suppresses the secretion of cholesterol and phosphatidylcholine. In SCAP conditional KO mice, pyramidal neurons exhibited a higher number of synapses but smaller spine head diameter, indicating an immature phenotype of post-synaptic compartments. These mice also displayed a lack of mature pre-synaptic terminals, characterized by a decreased level of synaptosomal-associated protein 25 (SNAP-25), a key molecule involved in pre-synaptic vesicle release, and a reduced number of pre-synaptic docked vesicles (van Deijk et al., 2017). Besides SCAP, fatty acid binding protein 7 (FABP7), a protein related to fatty acids transport, is also involved in synaptic maturation. In the medial prefrontal cortex (mPFC), FABP7 preferentially localizes in astrocytes and oligodendrocyte progenitor cells, but not localizes in neurons and microglia cells. FABP7 KO mice exhibited reduced neuronal dendritic arborization and impaired synaptic plasticity (Ebrahimi et al., 2016). Particularly, mPFC pyramidal neurons revealed decreased dendritic branches and length, the extent of area covered by the dendritic arbor, synapse density, and excitatory synaptic transmission. Consistently, co-cultures of wild-type neurons with FABP7-deficient astrocytes also lead to a reduction in dendritic complexity and spine density, confirming that FABP7 KO astrocytes are unable to fully support dendritic arborization and maturation of cortical neurons. In summary, neuron-glia lipid metabolism is critical for synaptogenesis, and any factors that can potentially disturb neuron-glia lipid metabolism can also potentially affect proper synaptogenesis.

## Astrocytes Regulate Synaptogenesis of Many Types of Synapses

After the initial findings from Barres laboratory, astrocyte-derived synaptogenic factors have received significant attention, and various synaptogenic factors have been discovered, including thrombospondins (TSP), high endothelial venule protein (hevin), secreted protein acidic and rich in cysteine (SPARC), brain-derived neurotrophic factors (BDNF), transforming growth factor β (TGF β) and γ-protocadherin. Among these secreted factors, most molecules are critical for glutamatergic synapse formation and maturation, or both, with some of these factors also regulating gamma-aminobutyric acid (GABA)-ergic, cholinergic, and glycinergic synaptogenesis.

### Thrombospondins Regulate Excitatory Synapse Formation

The most well-studied molecule involved in facilitating glutamatergic synapse formation is TSP. TSPs are soluble oligomeric extracellular matrix proteins that modulate cell-cell or cell-matrix contact by binding to membrane receptors or matrix proteins and cytokines. There are five TSPs in mammals, with three (TSP-1/2/4) of them being expressed in the CNS. All five TSPs are encoded by different genes and can form trimeric proteins including TSP-1/2, or pentameric proteins including TSP-4. Application of purified TSP-1 in RGCs increases the number of synapses indicated by pre-synaptic markers and post-synaptic markers. The increase of synaptic proteins is not due to increased protein expression level but increased synaptic localization (Christopherson et al., 2005). Consistently, electron microscope measurements show that a TSP-1 present medium is necessary for normal synapse formation. Synapses induced by TSP-1 are ultrastructurally identical to the chemical synapses promoted by an astrocyte feeding layer, specifically when looking at the length or thickness of post-synaptic density protein between synapses, and the number of docked and total vesicles per synapse. Interestingly, TSP-1 can only promote pre-synaptic function but not post-synaptic α-amino-3-hydroxy-5-methyl-4-isoxazole propionic acid (AMPA) receptor synaptic insertion. The astrocyte feeding layer treatment enhances pre-synaptic uptake of the antibody to the synaptotagmin luminal domain but fails to enlarge the amplitude of miniature excitatory post-synaptic currents (mEPSCs) and glutamate-evoked currents. Accordingly, biochemical experiments revealed that the C terminal of TSP1 interacts with the astrocytic Pentraxin 3′ N terminal. This interaction blocks Pentraxin3’s ability in promoting AMPA receptor synaptic clustering. Because applying Pentraxin3 in culture alone results in an increased dendritic AMPA receptor content and mEPSCs, which can be abolished by additional TSP1 but not TSP1 N terminal un-containing fragments (Fossati et al., 2019). Further *in vivo* experiments discovered that TSP-1/2 double null mutations, but not TSP1- or TSP-2-deficient, lead to a ∼40% decrease in the puncta number of pre-synaptic marker synaptic vesicle glycoprotein 2 (SV2), which indicates the functional pre-synaptic active zone, suggesting a robust mutual compensation between TSP-1 and TSP-2. In summary, TSP1/2 initiate events relating to the establishment of pre- and post-synaptic specializations and are thus critical components in promoting astrocyte-induced synaptogenesis. Furthermore, questions have been raised: “How do TSP play roles in synaptogenesis? Is there any neuronal receptor for TSP?” Using domain structure analysis, Eroglu et al. (2009) identified a voltage-gated calcium channel (VGCC) auxiliary subunit α2δ-1 (Cacna2d-1) as the neuronal receptor for TSP mediated synaptogenesis and proved that the von Willebrand factor A (VWF-A) domain of α2δ-1 directly interacts with epidermal growth factor like (EGF-like) repeats of TSP. Using gabapentin to disturb TSP-1/2 and α2δ-1 interactions, excitatory synapse formation can be dramatically inhibited *in vivo* and *in vitro*. Interestingly, pharmacological blocking of VGCC conductance or disturbing VGCC membrane expression had no effects on TSP-induced synaptogenesis. Instead, Risher et al. (2018) demonstrated that α2δ-1 promotes synaptogenesis and spinogenesis through the post-synaptic Ras-related C3 botulinum toxin substrate 1 (Rac1), a small Rho GTPase mainly involving in the regulation of the actin cytoskeleton. A landmark study also demonstrated how the expression of astrocytic factors is tightly controlled during development stages. Astrocytic TSP-1/2 reaches peak expression during post-natal days 5–10 and decreases in adulthood (Christopherson et al., 2005). Correspondingly, synaptogenesis peaks ∼10 days after TSP-1/2 peak expression. Thus, TSP-1/2 through α2δ-1-Rac1 pathways regulate synaptogenesis in the developing brain. However, there is little known about how post-synaptic Rac1 can affect pre-synaptic protein localization and vesicle docking.

### Hevin/SPARC Control Excitatory and Cholinergic Synapse Formation

Besides the extracellular matrix protein TSP, Kucukdereli et al. (2011) found two more astrocyte-derived matricellular proteins that modulate glutamatergic synapse formation. Specifically, they found hevin promoting synaptogenesis and SPARC antagonizing synaptogenic effects produced by hevin. Hevin null mutant mice had a smaller number of excitatory synapses than SPARC null mutant mice. The reason that SPARC acts as a competitive inhibitor of hevin is due to their similar domain structures. Similar situations exist among retinocollicular synapses, and the mechanism underlying the hevin-mediated glia-neuron signaling pathway has been identified. Hevin is a glycoprotein localized to synaptic clefts, where it is also localized with pre-synaptic neurexin-1 alpha (NRX1α) and post-synaptic neuroligin-1 (NL1). Hevin serves to bridge NRX1α and NL1 simultaneously to catalyze the formation of *trans*-synaptic neurexin/neuroligin complexes and downstream signaling pathways in thalamocortical glutamatergic connections (Risher et al., 2014; Singh et al., 2016). To further elucidate downstream signaling of the complex, a recent study surprisingly found that neurexin-1/2/3 triple KO or neuroligin-1/2/3/4 KO mice are both fully responsive to hevin, indicating that a novel mechanism exists beyond neurexins and neuroligins (Gan and Sudhof, 2020). In the hippocampus, SPARC ablation causes decreased synapse formation and aberrant surface AMPARs accumulation as well as impaired synaptic plasticity (Jones et al., 2011). Further evidence proves that SPARC disrupts properties of neuronal β3-integrin complexes, which are coupled to AMPAR synaptic stabilization. Unlike hevin, SPARC is responsive in blocking the maturation of cholinergic synaptic terminals (Albrecht et al., 2012), and autonomously triggers synapse elimination (Lopez-Murcia et al., 2015). Thus, hevin boosts glutamatergic synapse formation by forming NRX1α-hevin-NL1 complexes or other unknown mechanisms, while SPARC inhibits glutamatergic and cholinergic synaptogenesis by disrupting the function of neuronal β3-integrin complexes and NRX1α-hevin-NL1 complex formation across different regions in the developing brain.

### Glypicans Boost Synapse Formation and Maturation

TSP-1/2 and hevin make remarkable contributions to the initial formation of AMPA receptor lacking synapses, while astrocyte conditioned medium (ACM) can boost forming functional synapses, indicating that other unknown molecules exist in initiating AMPARs synaptic trafficking and clustering. To narrow down potential candidates, Allen et al. (2012) carefully analyzed ACM components by conducting two-dimensional electrophoresis and affinity column fractionation, and found that glypican (Gpc) 4/6 strengthen synapses by recruiting AMPARs to the surface and forming clusters. Western blots show that Gpc4/6 recruit the GluR1, but not the other subtypes, to the synaptic membrane. *In vivo* data confirmed that Gpc4 KO leads to a 22% decrease of matured synapses in the hippocampal CA1 region with less effect on total synapse number, as Gpc4 null mice show significantly reduced co-localization of GluR1 and post-synaptic marker. A further study uncovered the signaling cascade that Gpc4 upregulates neuronal expression of pentraxin 1 (NP1), which controls AMPAR synaptic localization (Farhy-Tselnicker et al., 2017). This study also reported that Gpc4 induces NP1 pre-synaptic release via type 2a receptor protein tyrosine phosphatases (RPTPδ). RPTPδ binds GluR1 receptors and recruits more AMPARs to the post-synaptic membrane, further promoting the maturation of synapses Thus, astrocytic Gpc4 through pre-synaptic RPTPδ and NP1 boots synapse maturation. Similar to RPTPδ, leucine-rich repeat transmembrane proteins (LRRTMs) also bind to Gpc4 to regulate synapse formation (de Wit et al., 2013; Siddiqui et al., 2013; Ko et al., 2015). Recent work discovered that post-synaptic LRRTM3 and LRRTM4 interact simultaneously with pre-synaptic neurexins and PTPσ to induce pre-synaptic differentiation (Roppongi et al., 2020). These two pre-synaptic hubs are capable of working separately and parallelly in a context-dependent manner, highlighting the complexity of the molecular logic of synaptogenesis.

### TGFβ Promotes Excitatory and Inhibitory Synapse Formation

In addition to extracellular matrix proteins, extrinsic cytokine TGF-β released from astrocytes boosts glutamatergic and GABAergic synaptogenesis (Bae et al., 2011; Diniz et al., 2012, 2014a,2014b). Astrocytic TGF-β1 binds to synaptic or astrocytic TGF-β receptors to induce D-serine release, and then together with glutamates to initiate post-synaptic *N*-methyl-D-aspartate (NMDA) receptor-dependent synapse formation and maturation (Diniz et al., 2012, 2014a,2014b). Interestingly, TGF-β1-induced inhibitory synapse formation also requires the activation of post-synaptic Ca^2+/^calmodulin-dependent protein kinase II (CaMKII), meaning that glutamate and D-serine bind to post-synaptic NMDAR and initialize neuroligin2 clustering at GABAergic synapses. *In vivo* data finds that TGF-β1 promotes the formation of ultrastructural normal and functional synapses. In contrast, genetic or pharmacological inhibition of D-serine signaling prevents synaptogenic effects of TGF-β1, further confirming that D-serine mediates TGF-β1-induced synaptogenesis. Since TGF-β is secreted as a precursor protein that requires extracellular proteolytic activation, it is important to elucidate the activator of TGF-β1 in the future study.

### BDNF Facilitates GABAergic Synaptogenesis

An *in vivo* work from the vestibular system reported that BDNF works with neuronal tyrosine receptor kinase B (TrkB) regulates the growth and branching of GABAergic axons (Gomez-Casati et al., 2010). The BDNF was released from supporting cells which have many characteristics of glia including the expression of glial fibrillary acidic protein (GFAP). It has been discovered that GFAP-DN-erbB4 mice (expression of a dominant-negative erbB4 receptor under glial fibrillary acidic promoter) display a severe deficiency in synapse formation and maintenance. By post-natal day 21, biochemical staining data revealed that presumptive synaptic sites in the utricle were reduced to 5%, with the presence of fewer pre- and post-synaptic specializations. This phenotype is due to erbB4 deficiency-induced reduction of BDNF secretion, because (1) glia-erbB is critical for BDNF production, (2) GFAP-DN-erbB4 expresses a substantial reduction in BDNF mRNA level, and (3) loss of BDNF phenocopies GFAP-DN-erbB4 effects. Taken together, glia induce synapse formation through reciprocal signals between neurons and glia, including BDNF-TrkB and neuregulin 1 (NRG1)-erbB pathways. Besides GABAergic synaptogenesis, glia also promotes glycinergic synaptogenesis. For example, in developing spinal cord neuron cultures, glia-free conditions reduce the frequency of spontaneous glycinergic inhibitory post-synaptic currents (IPSCs) but not GABAergic IPSCs. Adding ACM can prevent this decrement, suggesting that glia-derived soluble molecules can enhance glycinergic synaptic transmission (Cuevas et al., 2005). One study shows that adding purified TSP-1 in culture increases the number of synapses with inhibitory glycine receptors (GlyRs); blocking β1-integrins can abolish TSP-1’s effects. This finding provides a potential pathway for explaining the mechanism of glia-induced glycinergic synaptogenesis (Hennekinne et al., 2013). It is important to recognize that this study was conducted on a mature neuron culture different from the developing context. Future studies may provide more evidence relating to the identity of glia-neuron factors and the corresponding signaling cascade in neurons.

In summary, astrocytic TSP-1/2 and its partner α2δ-1 bind to their neuronal receptors and initiate synaptogenesis, while the NRX1α-hevin-NL1 complex bridges pre-synaptic and post-synaptic cell adhesion proteins. Moreover, adhesion molecule γ-protocadherins (Garrett and Weiner, 2009) can form glia-neuron homophilic interactions to initiate neuronal synaptogenic signaling cascades. It is worth mentioning that the neuron-astrocyte signal changes with development. At embryonic stages, RGCs develop pre-synaptic terminals and dendrites intrinsically but cannot gain synaptic receptivity; by embryonic day 19, astrocytes and RGCs generate direct contacts, resulting in synaptic receptivity. This indicates that astrocytic soluble factors alone are insufficient in inducing synapse maturation at an early stage (Barker et al., 2008). Consistently, in *in vitro* neuron cultures, young hippocampal neurons fail to utilize ACM for synaptogenesis, but physical neuron-astrocyte contact can promote functional maturation (Hama et al., 2004). During gestation, a prominent type of neuronal-astrocytic contact is electrical synapses which herald the later chemical synapse establishment (Fischbach, 1972; Connors et al., 1983; Jabeen and Thirumalai, 2013, 2018), therefore, electrical gap junction proteins may play a role in boosting chemical synapse formation and sculpting neurotransmission during synaptic development (Baker and Macagno, 2014). Imaging and electrophysiological data demonstrated that electrical gap junctions bridge bidirectional astrocytic-neuronal ionic and metabolic signaling in the human fetal hippocampal formation (Rozental et al., 2001). Knocking down the gap junction protein innexin 1 during early embryonic development resulted in impaired neuronal excitatory synaptic strength in juveniles (Todd et al., 2010). Moreover, glia also control their nearby GABAergic axon specification through the gap junctions in *C. elegans* (Meng et al., 2016). Taken together, astrocytes mainly use membrane-bound proteins to promote synaptogenesis at embryonic stages, whereas post-natal neurons can sense soluble factors released from astrocytes.

## Astrocytic Factor Secretion Is Under Tight Control

Astrocytes secrete various synaptogenic factors and express different cell adhesion proteins to induce neuronal responses. This raises interesting questions: What are the mechanisms underlying the synthesis and release of glia synaptogenic factors? Are these mechanisms programed as an intrinsic property of astrocytes or triggered by extrinsic signals? Few studies address these questions directly, but some have provided hints that can potentially lead to an answer. For example, repeated *in vivo* hippocampal electroconvulsive seizures (ECSs) upregulate TSP-1 mRNA level and protein expression but not TSP-2/4. This upregulation is transient, lasting ∼2 h and returning to basal level 24 h after ECSs (Okada-Tsuchioka et al., 2014). *In vitro* experiments have shown that ATP participates in TSP-1 production and release (Tran and Neary, 2006). This treatment of ATP in cultured cortical astrocytes results in a significant increase in TSP-1 expression in a time- and concentration-dependent manner, which can be blocked by antagonists of type 1/type 2 purinergic receptor (Tran and Neary, 2006). In addition, TSP-1 expression can be inhibited by exposure to reactive oxygen species (Chen et al., 2011). This means that TSP-1 expression can be triggered by external stimulation, though it is unclear whether astrocytes sense stimulation directly or detect cues released by neurons to trigger intrinsic TSP-1 generation. On a similar note, there are pieces of evidence supporting the possibility that neuronal cues could control the release of astrocytic factors. Firstly, astrocytes express neurotransmitter receptors. Secondly, manipulating neuron activity or dark rearing postpones astrocyte structural maturation in the visual cortex (Muller, 1990). Thirdly, genetic and pharmacological silencing of neuronal glutamatergic signaling leads to immature astrocytes in terms of structure and protein level (Morel et al., 2014). From this information, it is likely that extrinsic neurotransmitters can temporally and spatially regulate the release of glia factors. By controlling synaptogenic factors and CAMs, studies further uncovered the intra-astrocyte processes to answer how synaptogenic factors are precisely delivered to target sites, given that astrocytes can wrap up to 140,000 synapses. Sakers presents evidence that *de novo* protein synthesis occurs in fine astrocytic processes and ribosomal proteins present adjacent to synapses (Sakers et al., 2017). These findings convey the possibility that local productions in fine astrocytic processes play a role in the spatial control of synaptogenesis.

## Astrocytes Are Heterogenous

Histologically, astrocytes have been studied as a group of homologous cells, and it was considered that the properties of astrocytes in different CNS regions were interchangeable. The heterogeneity of astrocytes has been unappreciated for a long time, even though astrocytes have exhibited their morphological heterogeneity since Ramón y Cajal’s study. In recent years, glia biologists have noticed the diversity of astrocytes from every aspect and are aware of the importance of studying astrocytes as a group of heterogeneous cells. Various approaches have been adopted to address the heterogeneity of astrocytes, such as RNA-seq, proteomic analyses, and flow cytometry (Chai et al., 2017; Morel et al., 2019). Also, genetic approaches (Chai et al., 2017; John Lin et al., 2017; Morel et al., 2017, 2019; Miller et al., 2019), including translational ribosome affinity purification, promoter fragment labeling, and interceptional strategy, have been employed in accessing specific astrocyte subpopulations from intact tissues and in characterizing cell morphology or intrinsic properties. Indeed, astrocytes show inter-regional and intra-regional diversity in terms of origination, morphology, gene expression profile, and functional properties (Prochiantz and Mallat, 1988; Miller and Szigeti, 1991; Miyamura et al., 1998; Khakh and Sofroniew, 2015; John Lin et al., 2017; Taheri et al., 2017; Seifert et al., 2018; Weise et al., 2018; Durkee and Araque, 2019; Khakh and Deneen, 2019; Zhou et al., 2019; Batiuk et al., 2020; Gomes et al., 2020; Pham et al., 2020; Westergard and Rothstein, 2020). Using single-cell RNA sequencing, five astrocyte subtypes have been identified across mouse forebrain regions (Batiuk et al., 2020). Common genes across these five subtypes include *Dbx2*, *Sox9*, and *Apoe*, indicating their conserved functions in neural patterning, astrocyte specification, and cholesterol synthesis and trafficking, respectively. Meanwhile, these subtypes could possess a different capacity in regulating neurogenesis and neuronal differentiation, since over 70 percent of enriched genes are expressed in only one subtype of astrocytes. Here, we review how the heterogeneity of astrocytes impacts synaptogenesis. Other aspects involving astrocyte heterogeneity have been reviewed recently (Ben Haim and Rowitch, 2017; Durkee and Araque, 2019; Siracusa et al., 2019; Westergard and Rothstein, 2020) and, therefore, will not be covered here. An early study shows that glial cells from striatal or mesencephalic regions have different abilities in dendritic arborization (Denis-Donini et al., 1984). Dopaminergic neurons co-cultured with mesencephalic glia exhibit a great number of highly arborized neurites, but only long thin neurites are present when co-culture with striatal glia. Similarly, hippocampal astrocytes can differentiate adult rat hippocampal neural stem cells into neurons, whereas spinal cord-derived astrocytes do not have such capacity (Song et al., 2002). These findings highlight the inter-regional heterogeneity that astrocytes display regarding their capacity for promoting stem cell differentiation and neurites formation. One potential explanation for this distinct capability is the diversity of the astrocyte gene expression profiles. Recent findings proved that astrocytes derived from different brain regions exhibit variations of synaptogenic potential due to heterogeneity of synaptogenic relevant gene-expression profiles (Buosi et al., 2018). Astrocytes derived from the same anatomical region display higher synaptogenic capacity compared to mismatched co-cultures (Morel et al., 2017), indicating that synaptogenic matching of ligand and receptor is region-specific. A transcriptome study confirmed that most astrocyte-enriched genes are differentially expressed between regions (Boisvert et al., 2018; Bayraktar et al., 2020), which is analogous to neuronal cell-subtypes. For future studies, it would be useful to understand what causes the divergent capability of synaptogenesis and to ask whether transcriptional regulation and neuron-derived signals play roles in regulating astrocyte diversity (Farmer et al., 2016; Huang et al., 2020).

## The Application of Astrocytes Derived From hiPSCs

Human induced pluripotent stem cell is an attractive tool used to model a variety of neurological disorders (Solis, 2016; Chen et al., 2021), shedding light on the possibility of establishing a patient-based therapy using their cells. One challenge with hiPSC is the time-consuming process of *in vitro* differentiation and limited consistency across different studies (Tcw et al., 2017), meaning a large emphasis must be placed on minimizing variability. The nature of astrocytes in synaptogenesis inspires the use of astrocytes in the promotion of synapse maturation in hiPSC-derived neurons. Studies have shown that mouse primary astrocytes can boost neural differentiation, proliferation, survival, dendritic complexity, and expression of functional channel receptors when co-cultured with neural progenitor cells (NPCs) derived from hiPSCs (Tang et al., 2013). Two months of co-culture is sufficient for hiPSC derived neurons to display action potential, evoked currents, and spontaneous synaptic transmissions. Interestingly, the primary mouse astrocytes display higher potency compared to GCM, implying that physical contacts of astrocyte-neuron are pivotal in advancing the functional development of NPCs.

A caveat is that mouse astrocytes are dramatically different from human astrocytes in terms of morphology, gene profile, and function, with human astrocytes displaying significantly higher complexity and diversity compared to mouse astrocytes (Zhang et al., 2016). Specifically, observations from postmortem human tissues revealed that protoplasmic astrocytes in the human brain are three times larger and elaborate primary processes ten times more than those in rodents. Moreover, the interlaminar astrocytes in cortical layer 1 exist uniquely in humans and primates, and human cortical layer 5–6 polarized astrocytes exhibit extreme long processes compared with their rodents counterparts (Oberheim et al., 2006). Considering these differences, it is obvious that astrocytes derived from humans are more suitable for answering the questions about astrocyte pathology in neurological disorders at molecular and functional levels. Utilizing antibody-targeted surface protein or immunopanning-based method, Barres lab compared human astrocyte transcriptome profiling and functions (Zhang et al., 2016). Transcriptome data showed that 52% of genes enriched in mice were also identified in humans, whereas only 30% of human enriched genes were also enriched in the mouse. Over 600 human astrocyte genes that were not found in mouse astrocytes, such as intracellular calcium release relevant genes ryanodine receptor 3 (*Ryr3*) and mouse AIDS-related virus integration site 1 (*MRVI1*), which cloud help in understanding the distinct calcium properties present in human and murine astrocytes. However, it is still unknown if distinct calcium properties will result in a different capacity in synaptogenesis promotion, as intracellular calcium wave takes the major position in regulating glial transmitter release. Recently, by using a depletion approach, one study proved that astrocyte lineage cells are essential for neural differentiation and synapse maturation (Klapper et al., 2019). However, isogenic human astrocyte feeder layers failed to induce the display of spontaneous electrical activity in hiPSC-derived neurons, whereas parallel primary cortical astrocytes from mice induced such activity (Lischka et al., 2018). Clearly, the life span between murine and human are hugely different, which leads to a prolonged culturing period required for a human astrocyte to maturate. Thus, these isogenic human astrocytes were not fully mature and lacked potency revealed by RNA-seq data. Moreover, the loss of an *in vivo* environment may also be a limiting factor that dampens human astrocyte maturation *in vitro*.

**FIGURE 2:**
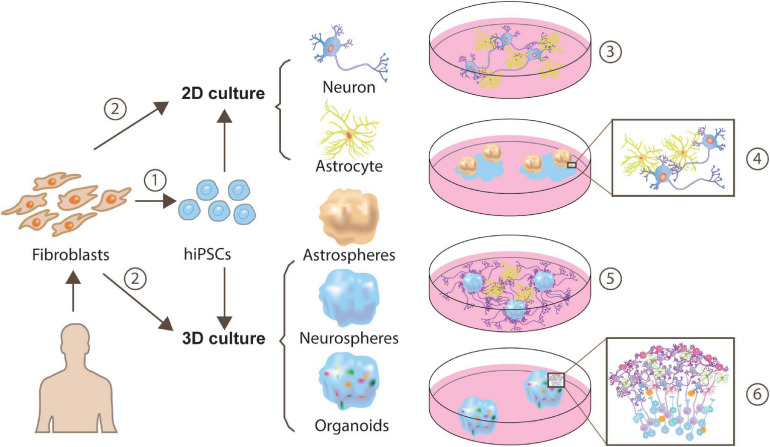
Approaches for generating human iPSC-derived neuron-astrocyte co-cultures. Human fibroblasts can be reprogrammed to a pluripotent state and yield cells exhibiting neuron or astrocyte features through monolayer generation (Tcw et al., 2017; Lundin et al., 2018) or 3D generation (Sloan et al., 2017) ➀. Besides, a more direct and time-saving way is direct conversion via transcription factors (TF), or expressing a minimal set of cell-lineage-specific TF in fibroblasts (Caiazzo et al., 2015) ➁. These neuron-astrocyte co-cultures can be assembled in multiple ways, such as 2-dimensional (2D) neuron-astrocyte co-culture ➂, plating astrospheres into 2D neuron culture➃ (Krencik et al., 2017), plating astrocytes into 3D neurospheres culture ➄ (Krencik et al., 2017) or cortical organoids ➅.

Fortunately, 3-dimensional (3D) neuron-astrocyte culturing is available (Pasca et al., 2015; Sloan et al., 2017; Agoglia et al., 2021) and can function to recapture key cellular and gene-expression features in the brain (see Figure 2). Cells in the 3D culture display self-organization and differentiation abilities, which recapitulate many aspects of human brain development (Pasca, 2019; Cherskov and Sestan, 2021). In terms of cell types, organoids contain neural lineage cells, including oligodendrocytes, neural stem cells, astrocytes, and neurons. Structurally, organoids display fluid-filled ventricle-like and layered structures. Neural lineage cells surround the ventricle-like structure forming a subventricular zone-like layer to produce neurons, therefore, giving rise to multiple layers. For example, pluripotent stem cell-derived human cortical spheroids include neurons from the most superficial to the deepest layer. Remarkably, the transcription map even matches *in vivo* fetal development. Imaging data demonstrated that neurons from human cortical spheroids are surrounded by astrocytes and form synapses, capturing processes including the interactions between the various neurons and glia in the brain. Further functional experiments proved that these synapses were electronically mature, exhibiting spontaneous activity and participating in-network activity. Absolutely, these properties of spheroids or organoids put them in a central position for a detailed questioning of development events, function and disease. Recently, the cortical organoid-based CRISPR-LICHT method (clustered regularly interspaced short palindromic repeats lineage tracing at cellular resolution in heterogeneous tissue) has been developed, which enables parallel loss-of-function screening in human organoids without the need of laboratory animals (Esk et al., 2020). Based on this high-throughput screening, Esk et al. (2020) revealed 25 out 173 candidate genes to be involved in known or unknown brain-size control associated pathway as well as potential mechanisms. This is a milestone achievement because it can help to get rid of slow and arduous animal loss-of-function experiments and recapture interactions among various cells. In addition, human organoid-based screening has unreplaceable merit compared to the use of animals, because genes associated with diseases can be expressed with different patterns across different species, and could therefore be problematic. Furthermore, Giandomenico et al. (2020) produced a new protocol to maintain cerebral organoids’ long-term viability through an increased organoid surface area. With the modification of embryoid body’s shape and size, their method successfully overcame the limitation involving the lack of nutrients and oxygen in interior organoid regions and enables the possibility of long-term organoid maintenance. Human organoids are a greatest tool in studying long-term processes of synaptogenesis *in vitro*. Together, organoids provide a more informative model for studying synaptogenesis and pathogenesis over extended periods among a large diversity of cell types, providing accessibility to the investigation of neuron-astrocyte interactions reminiscent of *in vivo* brain tissue.

## Conclusion and Future Perspectives

Astrocytes penetrate the entire CNS, they behave as an integral component of synapse and play essential roles in promoting synaptogenesis. Their synaptogenic capability displays regional-specificity given their heterogeneous identity. This laid the groundwork for investigating the diverse way in which astrocytes communicate with neurons. In this regard, several questions need to be addressed by future works, including: (1) how a subtype of astrocytes boost synapse formation and maturation in a specific region and at a particular brain developmental state, (2) does this signaling specificity exist in terms of the whole astrocyte or is it restricted to subcompartments, (3) can genetic tools be exploited to allow cell access for studying astrocyte subtype-specific functions. Moreover, human astrocytes exhibit higher complexity compared to rodents, studies from hiPSC derived organoids will take us one big step closer to understanding how the human brain is assembled in a relatively native environment.

## Author Contributions

LS and HL designed the conception and outline. LS researched and wrote the manuscript and made figures. HL supervised the writing of the manuscript. TZ, KF, and WC revised the manuscript. All authors contributed to the article and approved the submitted version.

## Conflict of Interest

WC is a scientific advisor, stockholder, and grantee of Focus-X Therapeutics, Inc. The remaining authors declare that the research was conducted in the absence of any commercial or financial relationships that could be construed as a potential conflict of interest.

## Abbreviations

2D: 2-dimensional
3D: 3-dimensional
5-HTR: 5-hydroxytryptamine receptor
ACM: astrocyte conditioned medium
AMPA: α-amino -3-hydroxy-5-methyl-4-isoxazole propionic acid
apoE: apolipoprotein E
AR: adenosine receptor
ATP: adenosine triphosphate
BDNF: brain-derived neurotrophic factors
bHLH-Zip: basic helix-loop-helix leucine zipper
CA: cornu ammonis
Cacna2d-1: voltage-gated calcium channel auxiliary subunit α2δ-1
CaMKII: Ca^2+^/calmodulin-dependent protein kinase II
CAMs: cell-adhesion molecules
CBR: cannabinoid receptor
cGMP: cyclic guanosine monophosphate
CNS: central nervous system
CRISPR-LICHT: clustered regularly interspaced short palindromic repeats lineage tracing at cellular resolution in heterogeneous tissue
D1R: dopamine receptor 1
ECSs: electroconvulsive seizures
EGF-like: epidermal growth factor like
FABP7: fatty acid binding protein 7
GABA: gamma-aminobutyric acid
GABA_a_R: inotropic gamma-aminobutyric acid receptor
GABA_b_R: metabotropic gamma-aminobutyric acid receptor
GCM: glia conditioned medium
GCY-8: receptor-type guanylate cyclase gcy-8
GFAP: glial fibrillary acidic protein
GFAP-DN-erbB4: expression of a dominant-negative erbB4 receptor under glial fibrillary acidic promoter
GlyRs: glycine receptors
Gpc: glypican
GPCRs: G protein-coupled receptors
GTP: guanosine-5 ′ -triphosphate
hevin: high endothelial venule protein
hiPSCs: human induced pluripotent stem cells
IPSCs: inhibitory post-synaptic currents
KCC-3: potassium chloride cotransporter 3
KO: knockout
LDL: light-density lipoprotein
LRRTMs: leucine-rich repeat transmembrane proteins
MAP2: microtubule-associate protein 2
mEPSCs: miniature excitatory post-synaptic currents
mGluR: metabotropic glutamate receptor
MOR: m-opioid receptor
mPFC: medial prefrontal cortex
MRVI1: mouse AIDS-related virus integration site 1
nAchR: nicotinic acetylcholine receptors
NL1: neuroligin-1B
NMDA: *N*-methyl -D-aspartate
NP1: pentraxin 1
NPCs: neural progenitor cells
NRG1: neuregulin 1
NRX1α: neurexin-1 alpha
P2XR: inotropic purinergic receptor
P2YR: metabotropic purinergic receptor
PAP: perisynaptic astrocytic process
PDGF: platelet-derived growth factor
PGC-1: peroxisome proliferator-activated receptor gamma coactivator 1
PSD: post-synaptic density protein
Rac1: Ras-related C3 botulinum toxin substrate 1
RGCs: retinal ganglion cells
ROS: reactive oxygen species
RPTPδ: type 2a receptor protein tyrosine phosphatases
Ryr3: ryanodine receptor 3
SAP: synapse-associated proteins
SCAP: SREBP cleavage-activating protein
SNAP-25: synaptosomal-associated protein 25
SPARC: secreted protein acidic and rich in cysteine
SPARCL1: SPARC-like protein 1
SREBP-1: sterol regulatory element-binding protein 1
SV2: synaptic vesicle glycoprotein 2
TGF: transforming growth factor
TrkB: tyrosine receptor kinase B
TSP: thrombospondin
VGCC: voltage-gated calcium channel
VGLUT1: vesicular glutamate transporter 1
VRAC: volume-regulated anion channel
VWF-A: von Willebrand factor A.
